# Emerging foodborne diseases.

**DOI:** 10.3201/eid0303.970304

**Published:** 1997

**Authors:** S F Altekruse, M L Cohen, D L Swerdlow

**Affiliations:** Centers for Disease Control and Prevention, Atlanta, GA 30333, USA. sba8@ciddbd1.em.cdc.gov

## Abstract

The epidemiology of foodborne diseases is rapidly changing. Recently described pathogens, such as Escherichia coli O157:H7 and the epidemic strain of Salmonella serotype Typhimurium Definitive Type 104 (which is resistant to at least five antimicrobial drugs), have become important public health problems. Well-recognized pathogens, such as Salmonella serotype Enteritidis, have increased in prevalence or become associated with new vehicles. Emergence in foodborne diseases is driven by the same forces as emergence in other infectious diseases: changes in demographic characteristics, human behavior, industry, and technology; the shift toward a global economy; microbial adaptation; and the breakdown in the public health infrastructure. Addressing emerging foodborne diseases will require more sensitive and rapid surveillance, enhanced methods of laboratory identification and subtyping, and effective prevention and control.


### Emerging Foodborne Diseases

**S.F. Altekruse, M.L. Cohen, and D.L. Swerdlow**
Centers for Disease Control and Prevention, Atlanta, Georgia, USA

The epidemiology of foodborne diseases is rapidly changing. Recently described pathogens, such as *Escherichia coli* O157:H7 and the epidemic strain of *Salmonella* serotype Typhimurium Definitive Type 104 (which is resistant to at least five antimicrobial drugs), have become important public health problems. Well-recognized pathogens, such as *Salmonella* serotype Enteritidis, have increased in prevalence or become associated with new vehicles. Emergence in foodborne diseases is driven by the same forces as emergence in other infectious diseases: changes in demographic characteristics, human behavior, industry, and technology; the shift toward a global economy; microbial adaptation; and the breakdown in the public health infrastructure. Addressing emerging foodborne diseases will require more sensitive and rapid surveillance, enhanced methods of laboratory identification and subtyping, and effective prevention and control.

Foodborne diseases have a major public health impact (Table 1; 1). In the United States, each year foodborne illnesses affect 6 to 80 million persons, cause 9,000 deaths, and cost an estimated 5 billion U.S. dollars (2). The epidemiology of foodborne diseases is rapidly changing as newly recognized pathogens emerge and well-recognized pathogens increase in prevalence or become associated with new food vehicles (Table 2). In addition to acute gastroenteritis, many emerging foodborne diseases may cause chronic sequelae or disability. Listeriosis, for example, can cause miscarriages (3) or result in meningitis in patients with chronic diseases (3). Toxoplasmosis is an important cause of congenital malformation (4), and *Escherichia coli* O157:H7 infection is a leading cause of hemolytic uremic syndrome, the most common cause of acute kidney failure in children in the United States (5). Salmonellosis can cause invasive disease (6) or reactive arthritis (7), and campylobacteriosis can lead to Guillain-Barré syndrome, one of the most common causes of flaccid paralysis in the United States in the last 50 years (8).

The factors contributing to the emergence of foodborne diseases are changes in human demographics and behavior, technology and industry, and international travel and commerce; microbial adaptation; economic development and land use; and the breakdown of public health measures (9). We describe selected foodborne pathogens, factors influencing their emergence, and possible controls.

Address for correspondence: Sean F. Altekruse, Mail Stop A-38, Centers for Disease Control and Prevention, Atlanta, GA, USA 30333; fax: 404-639-2212; e-mail: sba8@ciddbd1.em.cdc.gov.

Table 1. Estimated number of illnesses and deaths per year caused by infection with selected foodborne bacterial pathogens, United States (1-2)

| Pathogen | Cases ($10^3$) | Deaths ($10^3$) | Foods |
|---|---|---|---|
| *Campylobacter jejuni* | 4,000 | 0.2-1 | Poultry, raw milk, untreatedwater |
| *Salmonella* (nontyphoid) | 2,000 | 0.5-2 | Eggs, poultry, meat, fresh produce, other raw foods |
| *Escherichia coli* O157:H7 | 725 | 0.1-0.2 | Ground beef, raw milk, lettuce, untreated water, unpasteurized cider/apple juice |
| *Listeria monocytogenes* | 1.5 | 0.25-0.5 | ready-to-eat foods (e.g. soft cheese, deli foods, pâté) |
| *Vibrio* species | 10 | 0.05-0.1 | Seafood (e.g. molluscan, crustacean shellfish) raw, undercooked, cross-contaminated. |

Table 2. Selected outbreaks in the United States 1988–1997, associated with emerging foodborne pathogens and factors for the emergence of these pathogens

| Pathogen/outbreak | Location(s) | Year | Factors in emergence | Reference |
|---|---|---|---|---|
| Hepatitis A<br>Frozen strawberries | MI | 1997 | international travel and commerce<br>technology and industry | 28 |
| *Salmonella* Typhimurium DT 104<br>Farm visit | NE | 1996 | microbial adaptation | 48 |
| *Cyclospora cayetanensis*<br>Guatemalan raspberries | Multistate, Canada | 1996 | international travel and commerce | 25 |
| *Salmonella* Enteritidis PT 4<br>Egg-containing foods | CA | 1995 | international travel and commerce<br>technology and industry | 44 |
| *Salmonella* Enteritidis<br>Mass-distributed ice cream | Multistate | 1994 | technology and industry | 34 |
| Norwalk-like virus<br>Gulf Coast oysters | LA | 1994 | economic development and land use | 53 |
| *Escherichia coli* O157:H7<br>Fast-food chain hamburgers | Multistate | 1993 | technology and industry<br>breakdown of public health measures | 54 |
| *Escherichia coli* O157:H7<br>Raw apple cider | MA | 1991 | human demographics and behavior<br>technology and industry | 15 |
| *Vibrio cholerae* O1, El Tor<br>Thai coconut milk | MD | 1991 | international travel and commerce<br>human demographics and behavior | 39 |
| *Trichinella spiralis*<br>Undercooked pork | IA | 1990 | international travel and commerce<br>human demographics and behavior | 40 |
| *Salmonella* Chester<br>Sliced cantaloupe | Multistate | 1989 | international travel and commerce<br>human demographics and behavior | 22 |
| *Yersinia enterocolitica*<br>Pork chitterlings | GA | 1988 | human demographics and behavior | 41 |

### Selected Foodborne Pathogens of Public Health Importance

### *Salmonella* Serotype Enteritidis

Nontyphoidal salmonellosis is one of the most commonly reported infections in the United States. The doubling of salmonellosis incidence in the last two decades has accompanied modern food industries' centralized production and large-scale distribution. The most prevalent serotypes, *Salmonella* serotype Enteritidis (SE), *Salmonella* Typhimurium, and *Salmonella* Heidelberg, account for most human salmonellosis in the United States.

Some serotypes of *Salmonella*, such as *S.* Enteritidis, have specific animal reservoirs and are primarily transmitted by specific foods (10). Reflecting a worldwide trend, in the United States, the proportion of *Salmonella* isolates that were SE increased from 6% in 1980 to 25% in 1995 (Figure 1) (CDC, unpub. data). Between 1985 and 1991 in the United States, grade A shell eggs were implicated in 82% of SE outbreaks with known vehicles (10).

SE's ability to cause ovarian infections in egg-laying hens, thus contaminating the contents of intact shell eggs, has been important in the transmission of SE among humans and hens (11).

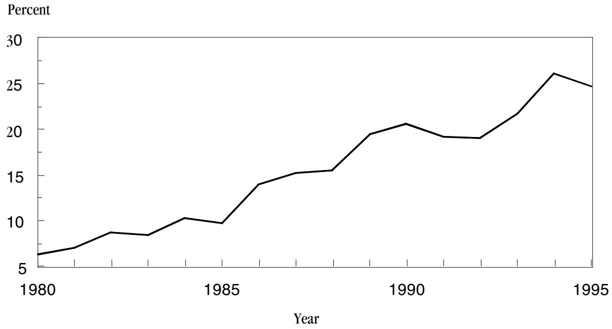

Figure 1. *Salmonella* serotype Enteritidis as a percentage of all *Salmonella* isolates reported in the United States, 1980–1995. Source: Centers for Disease Control and Prevention

SE can be transmitted vertically from breeding flocks to egg-laying hens, which in turn produce contaminated eggs (10,11). Once the organism is present in a flock, the infection is difficult to eliminate because transmission is sustained by environmental sources including rodents and manure.

### *Campylobacter jejuni*

*Campylobacter jejuni*, an emerging foodborne pathogen not recognized as a cause of human illness until the late 1970s, is now considered the leading cause of foodborne bacterial infection (12). An estimated four million *C. jejuni* infections occur each year in the United States; most sporadic infections are associated with improper preparation or consumption of mishandled poultry products (12). Incidence of campylobacteriosis is particularly high among young men. The high incidence of disease in this group may reflect poor food preparation skills (12). Most *C. jejuni* outbreaks, which are far less common than sporadic illnesses, are associated with consumption of raw milk or unchlorinated water (12).

The Guillain-Barré syndrome, an acute paralytic illness that may leave chronic deficits, may follow *Campylobacter* infections (8). In a multicenter study of 118 patients with Guillain-Barré syndrome in the United States, 36% had serologic evidence of *C. jejuni* infection in the weeks before neurologic symptoms developed(8).

### *E. coli* O157:H7

*E. coli* O157:H7 was first recognized as a human pathogen in 1982 when two outbreaks in the United States were associated with consumption of undercooked hamburgers from a fast-food restaurant chain (13). The pathogen has since emerged as a major cause of bloody and nonbloody diarrhea, causing as many as 20,000 cases and 250 deaths per year in the United States (2,5). Outbreaks have been reported in Canada, Japan, Africa, the United Kingdom, and elsewhere. In addition to causing bloody diarrhea, *E. coli* O157:H7 infection is the most common cause of the hemolytic uremic syndrome, the leading cause of acute kidney failure in children in the United States. The syndrome is associated with long-term complications; 3% to 5% of patients with hemolytic uremic syndrome die, and approximately 12% have sequelae including end-stage renal disease, hypertension, and neurologic injury (5). Consumption of ground beef (13), lettuce (14), raw cider (15), raw milk, and untreated water have been implicated in outbreaks, and person-to-person transmission is well documented (5).

### *Vibrio vulnificus*

In the late 1970s, *Vibrio vulnificus* was recognized to cause an usually severe syndrome of foodborne *V. vulnificus* infection called primary septicemia. *V. vulnificus* primary septicemia generally affects people with underlying disease, particularly liver disease. Patients become ill within 7 days after eating raw molluscan shellfish. Tracebacks implicate shellfish harvested from warm water areas. The symptoms may include shock and bullous skin lesions and may quickly progress to death. Most reported shellfish-associated *V. vulnificus* infections are fatal (16).

### *Listeria monocytogenes*

Since the early 1980s, foodborne transmission has been recognized as a major source of human listeriosis (3). Listeriosis can cause stillbirths, miscarriages, meningitis, or sepsis in immunocompromised hosts. Case-fatality rates as high as 40% have been reported during outbreaks (3). Outbreaks have been associated with ready-to-eat foods, including cole slaw, milk probably contaminated after pasteurization, pâté, pork tongue in jelly, and soft cheese made with inadequately pasteurized milk (3). The U.S. Department of Agriculture and U.S. Food and Drug Administration established zero tolerance policies for *L. monocytogenes* in foods in 1989. From 1989 to 1993, the food industry launched efforts to reduce *Listeria* contamination in processed foods, and dietary recommendations were established and publicized for persons at increased risk for invasive listeriosis. During this 4-year interval, the incidence of listeriosis declined by 40% in nine surveillance areas across the United States (17).

### Factors Contributing to the Emergence of Foodborne Diseases

### Human Demographics

Because of demographic changes in industrialized nations, the proportion of the population with heightened susceptibility to severe foodborne infections has increased. In the United States, a growing segment of the population is immunocompromised as a consequence of infection with human immunodeficiency virus (HIV), advancing age, or underlying chronic disease. Reported rates of salmonellosis, campylobacteriosis, and listeriosis were higher among HIV-infected persons than among those not infected with HIV (6). *Salmonella* (and possibly *Campylobacter*) infections are more likely to be severe, recurrent, or persistent in this population (6). Furthermore, extraintestinal disease caused by *Salmonella* and *L. monocytogenes* infection is more likely to be reported in HIV-infected persons than in the general population (6).

During the 20th century, the median age of the U.S. population steadily increased (18), a trend that is accelerating. (Figure 2). The elderly are at increased susceptibility to foodborne infections. In a series of seven SE outbreaks in nursing homes, for example, 10% of residents who became ill were hospitalized, and 7% died (19). In the general population, SE hospitalizations and death rates are much lower (10).

Advances in medical technology (e.g., organ transplantation and cancer therapy) have extended the life expectancy of persons with chronic diseases, thus increasing the proportion of the population with heightened susceptibility to severe foodborne illness. In the 1970s, for example, the 5-year survival rate for Hodgkin lymphoma was approximately 50%; by 1985, the 5-year survival rate approached 80%. The survival rate for all cancers combined also increased (20).

### Human Behavior

Changes in food consumption have brought to light unrecognized microbial foodborne hazards. Fresh fruit and vegetable consumption, for example, has increased nearly 50% from 1970 to 1994 (21). Fresh produce is susceptible to contamination during growth, harvest, and distribution. The surface of plants and fruits may be contaminated by human or animal feces. Pathogens on the surface of produce (e.g., melons) can contaminate the inner surface during cutting and multiply if the fruit is held at room temperature (22). In the United States from 1990 to 1997, increased consumption of fresh produce may have contributed to a series of foodborne outbreaks associated with foods such as sliced cantaloupe (22), green onions (23), unpasteurized cider (15), fresh squeezed orange juice (24), lettuce (14), raspberries (25), alfalfa sprouts (26), sliced tomatoes (27), and frozen strawberries (28).

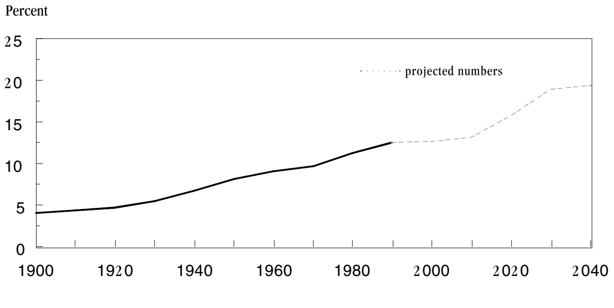

Figure 2. Percentage of U.S. population over 65 years of age, 1990–2040 (projected). Source: U.S. Bureau of Census

The percentage of spending on food eaten away from home has increased during recent decades (29). Fast-food restaurants and salad bars were rare 50 years ago but are primary sites for food consumption in today's fast-paced society (29). Outbreaks outside the home account for almost 80% of reported outbreaks in the United States in the 1990s (30). This figure may reflect the fact that these outbreak settings are most likely to be recognized and, therefore, reported to health officials; however, such food venues may also contribute to foodborne disease. Through practices such as the pooling of eggs, holding of hazardous foods at temperatures that permit amplification of low-dose pathogens, incomplete cooking of foods such as hamburgers, and cross-contamination of cooked foods.

Behavioral changes leading to foodborne infections are further complicated by decreased opportunities for food safety instruction both in school and at home. Health educators in secondary schools emphasize prevention of other important health concerns (e.g., HIV infection, obesity) over consumer safety issues including food safety education (31). In addition, because of two-income families and increased eating away from home, fewer opportunities may exist to pass food safety information from parent to child (29).

### Changes in Industry and Technology

The trend toward greater geographic distribution of products from large centralized food processors carries a risk for dispersed outbreaks. When mass-distributed food products are intermittently contaminated or contaminated at a low level, illnesses may appear sporadic rather than part of an outbreak (32).

Industry consolidation and mass distribution of foods may lead to large outbreaks of foodborne disease. In 1985, an outbreak of salmonellosis associated with contaminated milk from a large midwestern dairy was estimated to have resulted in approximately 250,000 illnesses (33). A nationwide outbreak of *Salmonella* serotype Enteritidis of similar magnitude occurred in 1994 when ice cream premix was transported in tanker trucks that had not been thoroughly sanitized after transporting raw liquid egg (34).

The trend toward larger markets and consolidation of industry has exacerbated the SE problem in another way. Changes in egg production have adversely affected infection control in poultry flocks (35). In 1945, a typical hen house contained 500 birds. By 1995, many houses contained 100,000 hens, and multiple houses were often linked by common machinery (35), resulting in large flocks with common risk profiles. Large-scale distribution of shell eggs from infected flocks has caused outbreaks in which contaminated eggs were distributed in many states over a period of months (10).

### Changes in Travel and Commerce

International travel has increased dramatically during the 20th century. Five million international tourist arrivals were reported worldwide in 1950, and the number is expected to reach 937 million by 2010 (Figure 3; 36). Travelers may become infected with foodborne pathogens uncommon in their nation of residence, thus complicating diagnosis and treatment when their symptoms begin after they return home. In 1992, for example, an outbreak of cholera caused 75 illnesses in international airline passengers; 10 persons were hospitalized, and one died (37). Pathogens may also be carried home to infect nontravelers. (38).

As the diversity of foods in the marketplace has increased, illnesses have been associated with internationally distributed foods. In 1992, an outbreak of epidemic cholera in Thai immigrants living in Maryland was caused by coconut milk imported from Thailand (39). In 1996, 1,465 cases of infection with *Cyclospora cayetanensis* were reported by 20 states, the District of Columbia, and two Canadian provinces. The investigation implicated raspberries from Guatemala (25).

In the mid-1990s, half to one and one-half million immigrants were admitted to the United States each year. Some reports of foodborne illnesses involve transmission by foods consumed primarily by immigrant groups. Outbreaks of

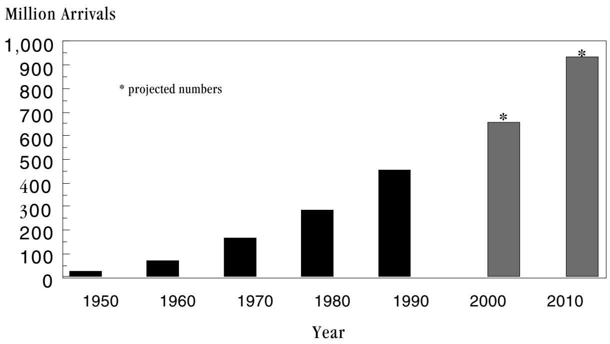

Figure 3. Global international tourist arrivals, in millions per year. 1950–2010 (projected). Source: World Tourism Organization trichinosis have become relatively rare in the United States because cooking pork thoroughly has become a widespread cultural practice. An exception occurred in 1990, when Laotian immigrants in Iowa prepared and ate undercooked pork, a traditional food, in celebration of a wedding (40). Other reports involve foods consumed by ethnic populations. *Yersinia enterocolitica* outbreaks are also rare, but several have occurred in inner-city African-American communities and were associated with preparation and consumption of pork intestines (41). The epidemiology of human brucellosis in California has shifted from an occupational disease of animal husbandry to a foodborne disease most frequently affecting Hispanics who often while abroad consume raw milk and cheeses made with raw milk (42).

### Microbial Adaptation

### Environmental Conditions

Natural selection is a key process in the emergence of pathogens (11). Microbes adapt to have an advantage in unfavorable environments (e.g., heat and acidity) (43). SE phage type (PT) 4 may have developed traits that enable it to rapidly replace closely related SE phage types in egg-laying poultry environments (43). During the 1980s, for example, SE PT 4 became the predominant phage type in humans and poultry in Europe and caused a marked increase in human illnesses. SE PT 4 was rare in the United States before 1994 when SE PT 4 emerged in southern California, resulting in a fivefold increase in reported human SE infection (44). In 1995, a similar increase in SE PT 4 infection was reported in Utah.

### Antimicrobial Resistance

The therapeutic use of an antimicrobial agent, in human or animal populations, creates a selective pressure that favors survival of bacterial strains resistant to the agent. Antimicrobial-resistant strains of *Salmonella* have become increasingly prominent (45). In the United States, the percentage of antimicrobial resistant *Salmonella* infections increased from 17% of isolates in the late 1970s to 31% in the late 1980s (45). Compared with patients with susceptible infections, patients with antimicrobial-resistant infections are more likely to require hospitalization and to be hospitalized for longer periods (45).

During the 1990s, *Salmonella* serotype Typhimurium Definitive Type 104 (DT 104) emerged in the United Kingdom. By 1995, DT 104 was the second most common cause of human salmonellosis in England and Wales; more than 3,800 isolates were reported from humans in that year alone (46). Ninety percent of all DT 104 isolates were resistant to ampicillin, chloramphenicol, streptomycin, sulphonamides, and tetracycline (R-type ACSSuT). Strains of *S.* Typhimurium DT 104 with resistance to trimethoprim and ciprofloxacin have also emerged in the United Kingdom. Surveillance for DT 104 infections in the United Kingdom indicated high hospitalization and mortality rates with this organism compared with infections caused by other *Salmonella* serotypes. In one study more than 10% of patients with multidrug-resistant DT 104 infection required hospitalization, and more than 3% died (46). In the United Kingdom, illness has been associated with contact with farm animals and with consumption of foods, including beef, pork sausages, and chicken. The organism has been isolated primarily from cattle but also from poultry, sheep, and pigs (46).

*S.* Typhimurium DT 104 is rapidly emerging in the United States. A study of *S.* Typhimurium isolates from the Pacific Northwest indicated that 43% of human isolates obtained in 1994 had R-type ACSSuT compared with 4% in 1989 (47). Among cattle isolates of *S.* Typhimurium from the Pacific Northwest obtained before 1986, none had this R-type, compared with 13% of isolates obtained between 1986 and 1991, and 64% of isolates obtained between 1992 and 1995 (47). When 25 isolates from either human or cattle sources in the Pacific Northwest with R-type ACSSuT were phage-typed, all were *S.* Typhimurium DT 104 (47). The characteristic resistance pattern (R-type ACSSUT) was present in 32% of human *S.* Typhimurium isolates tested in 1996. The first confirmed outbreak of *S.* Typhimurium DT 104 in the United States occurred in Nebraska in 1996 (48).

Another example of an antimicrobial-resistant foodborne pathogen is fluoroquinolone-resistant *C. jejuni*, which has increased in Europe since the early 1990s (49). The emergence of human infections has been temporally associated with the approval of fluoroquinolones for veterinary use in Europe.

### Economic Development and Land Use

In the United States, food animals generate over 1.6 billion tons of manure per year (50). On large-scale production facilities, manure disposal is a growing problem. Without disposal, manure may serve as a reservoir for *Salmonella*, *C. jejuni*, and other farm pathogens.

The shift from a cold season oyster harvest in the Gulf of Mexico to a year-round harvest (51) is a change in resource use associated with the emergence of *V. vulnificus*. *V. vulnificus* primary septicemia has a summer seasonality, and most tracebacks implicate raw oysters from the Gulf of Mexico (52). Although the annual oyster harvest from the U.S. Gulf of Mexico has not changed since the 1930s, the percentage of oysters harvested during summer months increased from 8% of the annual harvest in 1970 to 30% in 1994 (51). The disposal of feces in oyster beds by oyster harvesters with gastroenteritis has been implicated in shellfish-associated Norwalk-like virus outbreaks, including one in Louisiana in 1994 (53).

### Breakdown of the Public Health Infrastructure

Many public health agencies operate with extremely limited resources. The consequent breakdown in public health infrastructure increases the potential for underreporting of foodborne infections (54). In the mid-1990s, for example, 12 states had no personnel dedicated to foodborne disease surveillance (54), largely because of budget restrictions at the state and local levels. When the infrastructure for infectious diseases surveillance is compromised, recognition of outbreaks is jeopardized (9,54).

### Prevention and Control

The prevention of foodborne disease depends on careful food production, handling of raw products, and preparation of finished foods.

Hazards can be introduced at any point from farm to table. Technologies are available to prevent many foodborne illnesses. Just as the 20th century's revolution in food sanitation and hygiene (including refrigeration, chlorination of drinking water, pasteurization of milk, and shellfish monitoring) was a consequence of applied technologies, industrial engineering can hold the key to food safety in the 21st century. Among technologies that merit evaluation are chlorination of drinking water sources for food animals (55); sanitary slaughter and processing of meat (56), poultry (56), and seafood (57); irradiation (58); and other microbial reduction steps for raw agricultural commodities.

When monitoring and control technologies are systematically applied to food production to prevent foodborne illnesses, the program is said to use a Hazard Analysis Critical Control Point (HACCP) process (56,57). Such programs require food industries to identify points in food production where contamination may occur and target resources toward processes that may reduce or eliminate foodborne hazards. In the 1990s, programs were implemented by meat (56), poultry (56), and seafood (57) industries and federal regulatory agencies. In these programs, industry takes the lead for the control of foodborne hazards, and regulatory agencies maintain oversight.

Preparers of meals are the last critical control point before foods reach the table. Interventions to promote safe food preparation practices are needed (59). Food preparers can reduce the risk of foodborne diseases with a few practical food-handling precautions. Thorough heating of potentially hazardous foods kills pathogens, and refrigeration prevents their multiplication. Cross-contamination of foods can be avoided by separating cooked and raw foods and preventing contamination of cooked foods by drippings from raw foods. Foodworkers should wash hands, cutting boards, and contaminated surfaces as warranted to prevent cross-contamination. Consumers can reduce the risk of foodborne infections by avoiding high-risk foods, such as runny eggs, hamburgers that are pink at the center, and raw shellfish.

Each link in the production, preparation, and delivery of food can be a hazard to health. While technologies designed to improve the safety of the food supply hold promise, changes in food processing, products, practices, and people will continue to facilitate the emergence of foodborne pathogens into the next century. Foodborne disease surveillance provides a basis for detecting disease and identifying points at which new strategies are needed to protect the food supply.

Dr. Altekruse is a veterinary epidemiologist with the U.S. Food and Drug Administration, assigned as a liaison officer to CDC's Foodborne and Diarrheal Diseases Branch. His research activities include surveillance for noncholera *Vibrio* infections.

### References

1. Helmick CG, Griffin PM, Addiss DG, Tauxe RV, Juranek DD. Infectious diarrheas. In: Everhart JE, editor. Digestive diseases in the United States: epidemiology and impact. Bethesda (MD): National Institutes of Health, 1994; Publication No. 94-1447. p. 85-120.
2. Council for Agricultural Science and Technology. Foodborne pathogens: risks and consequences. Ames (IA): The Council; 1994. Task Force Report No. 122.
3. Schuchat A, Swaminathan B, Broome CV. Epidemiology of human listeriosis. Clin Microbiol Rev 1991;4:169-83.
4. Kapperud G, Jenum PA, Stray-Pedersen B, Melby KK, Eskild A, Eng J. Risk factors for *Toxoplasma gondii* infection in pregnancy. Am J Epidemiol 1996;144:405-12.
5. Boyce TG, Swerdlow DL, Griffin PM. *Escherichia coli* O157:H7 and the hemolytic-uremic syndrome. N Engl J Med 1995;333:364-8.
6. Altekruse SF, Hyman FH, Klontz KC, Timbo BT, Tollefson LK. Foodborne bacterial infections in individuals with the human immunodeficiency virus. South Med J 1994;87:169-73.
7. Swerdlow DL, Lee LA, Tauxe RV, Bean NH, Jarvis JQ. Reactive arthropathy following a multistate outbreak of *Salmonella typhimurium* infections [abstract]. Proceedings of the 30th Interscience Conference on Antimicrobial Agents and Chemotherapy; 1990 Oct 21-24; Atlanta, GA. Washington (DC): American Society for Microbiology; 1990. Abstract No. 916.
8. Mishu B, Ilyas AA, Koski CL, Vriesendorp F, Cook SD, Mithen FA, et al. Serologic evidence of previous *Campylobacter jejuni* infection in patients with the Guillain-Barré syndrome. Ann Intern Med 1993;118:947-53.
9. Institute of Medicine. Emerging infections: microbial threats to health in the United States. Washington (DC): National Academy Press; 1992.
10. Mishu B, Koehler J, Lee LA, Rodrigue D, Hickman-Brenner F, Blake P, et al. Outbreaks of *Salmonella enteritidis* infections in the United States, 1985-1991. J Infect Dis 1994;169:547-52.
11. Snoeyenbos GH, Smyzer CF, Van Roekel H. *Salmonella* infections of the ovary and peritoneum of chickens. Avian Dis 1969;13:668-70.

12. Tauxe RV. Epidemiology of *Campylobacter jejuni* infections in the United States and other industrialized nations. In: Nachamkin I, Tompkins S, Blaser M, editors. *Campylobacter jejuni*: Current Status and Future Trends. Washington (DC): American Society for Microbiology; 1992.
13. Riley LW, Remis RS, Helgerson SD, McGee HB, Wells JB, Davis BR, et al. Hemorrhagic colitis associated with a rare *Escherichia coli* serotype. N Engl J Med 1983;308:681-5.
14. Ackers M, Mahon B, Leahy E, Damrow T, Hutwagner L, Barrett T, et al. An outbreak of *Escherichia coli* O157:H7 infections associated with leaf lettuce consumption, Western Montana [abstract]. Proceedings of the 36th Interscience Conference on Antimicrobial Agents and Chemotherapy; 1996 Sep 15-18; New Orleans, LA. Washington (DC): American Society for Microbiology; 1996:257. Abstract No. K43.
15. Besser RE, Lett SM, Weber T, Doyle MP, Barrett TJ, Wells JG, et al. An outbreak of diarrhea and hemolytic uremic syndrome from *Escherichia coli* O157:H7 in fresh pressed apple cider. JAMA 1993;269:2217-20.
16. Blake PA, Merson MH, Weaver RE, Hollis DG, Heublin PC. Disease caused by a marine *Vibrio*. Clinical characteristics and epidemiology. N Engl J Med 1979;300:1-5.
17. Tappero JW, Schuchat A, Deaver KA, Mascola L, Wenger JD. Reduction in the incidence of human listeriosis in the United States. Effectiveness of prevention efforts? JAMA 1995;273:1118-22.
18. Bureau of the Census, U.S. Department of Commerce. 1990 Census of population. General population characteristics. Washington (DC): U.S. Government Printing Office; 1990; CP-1-1 20240.
19. Levine WC, Smart JF, Archer DL, Bean NH, Tauxe RV. Foodborne disease outbreaks in nursing homes, 1975 through 1987. JAMA 1991;266:2105-9.
20. Miller BA, Ries LAG, Hankey BF, Kosary CL, Harras A, Devesa SS, Edwards BK, editors. SEER Cancer Statistics Review 1973-1990. Bethesda (MD): National Cancer Institute; 1991. NIH Publication 93-789.
21. Bureau of the Census, U.S. Department of Commerce. Per capita utilization of selected commercially produced fresh fruits and vegetables: 1970 to 1994. Statistical Abstract of the United States, 116th Edition. Washington (DC): U.S. Government Printing Office; 1996. p. 148.
22. Reis AA, Zaza S, Langkop C, Tauxe RV, Blake PA. A multistate outbreak of *Salmonella chester* linked to imported cantaloupe [abstract]. Proceedings of the 30th Interscience Conference on Antimicrobial Agents and Chemotherapy; 1990 Oct 21-24; Atlanta, GA. Washington (DC): American Society for Microbiology; 1990:38.
23. Cook KA, Boyce T, Langkop C, Kuo K, Swartz M, Ewert D, et al. Scallions and shigellosis: a multistate outbreak traced to imported green onions [abstract]. Proceedings of the 44th Annual Conference of the Epidemic Intelligence Service; 1995 March 27–31; Atlanta, GA. Atlanta (GA): Centers for Disease Control and Prevention; 1995:36.
24. Cook KA, Swerdlow D, Dobbs T, Wells J, Puhr N, Hlady G, et al. Fresh-squeezed *Salmonella*: an outbreak of *Salmonella hartford* associated with unpasteurized orange juice—Florida [abstract]. Proceedings of the 45th Annual Epidemic Intelligence Service Conference; 1990 Apr 22-26; Atlanta, GA. Atlanta (GA): Centers for Disease Control and Prevention; 1996:38.
25. Herwaldt BL, Ackers M-L, and the Cyclospora Working Group. An outbreak in 1996 of cyclosporiasis associated with imported raspberries. N Engl J Med 1997;336:1548-56.
26. Mahon BE, Ponka A, Hall W, Komatsu K, Dietrich SE, Siitonen A, et al. An international outbreak of *Salmonella* infections caused by alfalfa sprouts grown from contaminated seeds. J Infect Dis 1997;175:876-82.
27. Wood RC, Hedberg C, White K, MacDonald K, Osterholm M. A multi-state outbreak of *Salmonella javiana* infections associated with raw tomatoes [abstract]. Proceedings of the 40th Annual Conference of the Epidemic Intelligence Service; 1991 April 8–12; Atlanta, GA. Atlanta (GA): Centers for Disease Control and Prevention; 1991:69.
28. Hepatitis A associated with consumption of frozen strawberries—Michigan, March 1997. MMWR Morb Mortal Wkly Rep 1997;46:288,295.
29. Manchester A, Clauson A. 1994 spending for food away from home outpaces food at home. Food Review 1995;18:12-5.
30. Bean NH, Goulding JS, Lao C, Angulo FJ. Surveillance for foodborne disease outbreaks—United States, 1988-1991. CDC Surveillance Summaries, October 25, 1996. MMWR Morb Mortal Wkly Rep 1996;45(SS-5):1-66.
31. Collins JL, Small ML, Kann L, Pateman BC, Gold RS, Kolbe LJ. School health education. J Sch Health 1995;65:302-11.
32. Killalea D, Ward LR, Roberts D, de Louvois J, Sufi F, Stuart JM, et al. An outbreak of *Salmonella agona* infection in England and the United States caused by contamination of a ready-to-eat savoury snack. BMJ 1996;313:1105-7.
33. Ryan CA, Nickels MK, Hargrett-Bean NT, Potter ME, Endo T, Mayer L, et al. Massive outbreak of antimicrobial-resistant salmonellosis traced to pasteurized milk. JAMA 1987;58:3269-74.
34. Hennessy TW, Hedberg CW, Slutsker L, White KE, Besser-Wiek JM, Moen ME, et al. A national outbreak of *Salmonella enteritidis* infections from ice cream. N Engl J Med 1996;334:1281-6.
35. Bell D. Forces that have helped shape the U.S. egg industry: the last 100 years. Poultry Tribune. 1995:30-43.
36. Paci E. Exploring new tourism marketing opportunities around the world. Proceedings of the Eleventh General Assembly of the World Tourism Organization; 1995 Oct 15; Cairo, Egypt. Cairo, Egypt: World Tourism Organization; 1995.
37. Eberhart-Phillips J, Besser RE, Tormey MP, Feikin D, Araneta MR, Wells J, et al. An outbreak of cholera from food served on an international aircraft. Epidemiol Infect 1996;116:9-13.
38. Finelli L, Swerdlow D, Mertz K, Ragazzoni H, Spitalny K. Outbreak of cholera associated with crab brought from an area with epidemic disease. J Infect Dis 1992;166:1433-6.
39. Taylor JL, Tuttle J, Pramukul T, O'Brien K, Barrett TJ, Jolbitado B, et al. An outbreak of cholera in Maryland associated with imported commercial coconut milk. J Infect Dis 1993;167:1330-5.
40. Stehr-Green JK, Schantz PM. Trichinosis in Southeast Asian refugees in the United States. Am J Public Health 1986;76:1238-9.

41. Lee LA, Gerber AR, Lownsay DR, Smith JD, Carter GP, Puhr ND, et al. *Yersinia enterocolitica* O:3 infections in infants and children associated with household preparation of chitterlings. N Engl J Med 1990;322:984-7.
42. Chomel BB, DeBess EE, Mangiamele DM, Reilly KF, Farver TB, Sun RK, et al. Changing trends in the epidemiology of human brucellosis in California from 1973 to 1992: a shift toward foodborne transmission. J Infect Dis 1994;170:1216-23.
43. Humphrey TJ, Slater E, McAlpine K, Rowbury RJ, Gilbert RJ. *Salmonella enteritidis* phage type 4 isolates more tolerant of heat, acid, or hydrogen peroxide also survive longer on surfaces. Appl Environ Microbiol 1995;61:3161-4.
44. Passaro DJ, Reporter R, Mascola L, Kilman L, Malcolm G, Rolka H, et al. Epidemic illness caused by *Salmonella enteritidis* infection in Los Angeles County: the predominance of phage type 4. West J Med 1996;165:126-30.
45. Lee LA, Puhr ND, Maloney EK, Bean NH, Tauxe RV. Increase in antimicrobial-resistant *Salmonella* infections in the United States, 1989-1990. J Infect Dis 1994;170:18-34.
46. Threlfall EJ, Hampton MD, Schofield SL, Ward LR, Frost JA, Rowe B. Epidemiological application of differentiating multiresistant *Salmonella typhimurium* DT 104 by plasmid profile. Commun Dis Rep CDR Rev 1996;6:R155-9.
47. Besser TE, Goldoft M, Gay CC. Emergence of *Salmonella typhimurium* DT 104 in humans and animals in the Pacific Northwest. Proceedings of the 51st Annual Northwestern International Conference on Diseases in Nature Communicable to Man; Seattle, WA; 1996 Aug 11–14. Hosted by Washington State Department of Health.
48. Multidrug-resistant *Salmonella* Serotype Typhimurium—U.S., 1996. MMWR Morb Mortal Wkly Rep 1997;46:308-10.
49. Endtz HP, Ruijs GJ, van Klingeren B, Jansen WH, van der Reyden T, Mouton RP. Quinolone resistance in *Campylobacter* isolated from man and poultry following the introduction of fluoroquinolones in veterinary medicine. J Antimicrob Chemother 1991;27:199-208.
50. Natural Resources Conservation Service, U.S. Department of Agriculture, Animal manure management. Washington (DC): U.S. Department of Agriculture; 1995; Issue Brief 7. p. 1-6.
51. National Marine Fisheries Service. Monthly oyster landings by region. Silver Spring (MD): U.S. Department of Commerce, National Oceanic and Atmospheric Administration; 1995.
52. Centers for Disease Control and Prevention. *Vibrio vulnificus* infections associated with eating raw oysters—Los Angeles. MMWR Morb Mortal Wkly Rep 1996;45:621-4.
53. Kohn MA, Farlet TA, Ando T, Curtis M, Wilson SA, Jin Q, Monroe SS, et al. An outbreak of Norwalk virus gastroenteritis associated with eating raw oysters. Implications for maintaining safe oyster beds. JAMA 1995;273:466-71.
54. Berkelman RL, Bryan RT, Osterholm MT, LeDuc JW, Hughes JM. Infectious disease surveillance: a crumbling foundation. Science 1994;264:368-70.
55. Kapperud G, Skjerve E, Hauge K, Lysaker A, Aalmen I, Ostroff SM, et al. Epidemiological investigation of risk factors for *Campylobacter* colonization in Norwegian broiler flocks. Epidemiol Infect 1993;111:45-55.
56. Pathogen Reduction; Hazardous Analysis and Critical Control Point (HACCP) Systems, 9 C.F.R. 304, 308, 310, 320, 327, 381, 416, 417 (1996).
57. Procedures for the Safe and Sanitary Processing and Importing of Fish and Fishery Products, 21 C.F.R. Pt. 123, 1240 (1995).
58. Steele JH, Engel RE. Radiation processing of foods. J Am Vet Med Assoc 1992;201:1522-9.
59. Altekruse SF, Street DA, Fein SB, Levy AS. Consumer knowledge of foodborne microbial hazards and food-handling practices. Journal of Food Protection 1996;59:287-94.
